# Impact of Propofol Sedation upon Caloric Overfeeding and Protein Inadequacy in Critically Ill Patients Receiving Nutrition Support

**DOI:** 10.3390/pharmacy9030121

**Published:** 2021-07-01

**Authors:** Roland N. Dickerson, Christopher T. Buckley

**Affiliations:** 1 College of Pharmacy, University of Tennessee Health Science Center, Memphis, TN 38163, USA; 2 College of Pharmacy, Union University, Jackson, TN 38305, USA; cbuckley@uu.edu

**Keywords:** propofol, enteral nutrition, parenteral nutrition, fat emulsions, critical illness, nutritional requirements, hypnotics and sedatives, food–drug interactions

## Abstract

Propofol, a commonly used sedative in the intensive care unit, is formulated in a 10% lipid emulsion that contributes 1.1 kcals per mL. As a result, propofol can significantly contribute to caloric intake and can potentially result in complications of overfeeding for patients who receive concurrent enteral or parenteral nutrition therapy. In order to avoid potential overfeeding, some clinicians have empirically decreased the infusion rate of the nutrition therapy, which also may have detrimental effects since protein intake may be inadequate. The purpose of this review is to examine the current literature regarding these issues and provide some practical suggestions on how to restrict caloric intake to avoid overfeeding and simultaneously enhance protein intake for patients who receive either parenteral or enteral nutrition for those patients receiving concurrent propofol therapy.

## 1. Introduction

Propofol is a commonly used intravenous sedative for ventilator-dependent patients. Its advantage over other sedative agents, such as benzodiazepines, is in its rapid onset and short half-life which allows for daily awakening and spontaneous breathing trials. The use of propofol is recommended by the Brain Trauma Foundation for the treatment of elevated intracranial pressure [1] and sometimes prolonged continuous large doses are required. Prolonged use of large doses can be problematic in the nutritional management of these patients since propofol is formulated in a 10% lipid emulsion as either soybean oil in the United States or available in a mixed oil formulation internationally. Due to the oil carrier solution for propofol, the intravenous infusion contains 1.1 kcal/mL. As a result, complications associated with caloric overfeeding, hypertriglyceridemia, and inadequate protein intake may occur for patients receiving propofol therapy [2]. The intent of this review is to examine caloric intake associated with propofol therapy and to describe some strategies to avoid overfeeding that will still meet the increased protein needs of critically ill patients who require enteral nutrition (EN) or parenteral nutrition (PN) therapy.

## 2. Methods

The search engines used for identifying pertinent articles for the literature review were PubMed, Embase, and Google scholar from their database inceptions to March 2021. The same search strategy was used for all databases using the following key word phrases: (propofol AND calories) and (propofol AND protein AND intake). Articles were restricted to the English language. Any duplicate articles obtained from the different databases were removed. Inclusion criteria were original research studies, observational case series, and abstracts from scientific meetings that have not yet been published as a full paper. References cited in narrative reviews regarding the caloric contribution of propofol and from the identified articles were screened for other potentially relevant articles. Studies that did not separate patients who received caloric intake from propofol from those who did not receive propofol were excluded. Case reports were also excluded.

## 3. Results

A total of fourteen research publications and abstracts published from 1995 to 2021 were obtained from the literature (Table 1) [3,4,5,6,7,8,9,10,11,12,13,14,15,16]. Five publications [4,7,9,12,14] were only available in abstract form with limited information. Most of the reports studied propofol use in a heterogenous intensive care unit (ICU) patient population; however, some studies were focused upon a select patient population such as trauma and neurosurgical patients [6,12,15], COVID-19 patients [7], or those receiving extracorporeal membrane oxygenation [10]. Five studies included patients who concurrently received propofol with either EN or PN, whereas the remaining studies only reported those who received concurrent EN with propofol therapy. Average caloric intake from propofol ranged from 60 kcals/d to 356 kcals/d; however, some studies reported considerable variability with intakes from propofol exceeding 500 kcals/d for some patients [5,6,9]. Propofol contributed to 5% to 24% of total caloric intake when combined with EN or PN (Table 1). Only eight studies [6,7,10,11,12,14,15,16] reported concurrent mean protein intakes, with most studies reporting intakes less than 1.2 g/kg/d. The highest reported mean protein intake was 1.5 ± 0.7 g/kg/d [6].

## 4. Discussion

The literature suggests that intravenous propofol infusion can significantly contribute to the overall caloric intake for the patient who receives EN or PN. This is particularly pertinent for the critically ill patients in the ICU with traumatic brain injury (TBI) who often receives prolonged propofol therapy at high doses to assist in the reduction in intracranial pressure, cerebral metabolic rate, and to improve cerebral perfusion pressure [1,17]. Additionally, because of its rapid onset and short half-life, it allows for frequent neurologic exams, which renders it a uniquely useful agent for patients with TBI. It is less likely to accumulate in the body during prolonged infusions such as benzodiazepines. However, aggressive dosing of intravenous lipid emulsion-based propofol therapy poses as significant risk factor for overfeeding complications in patients who receives concurrent EN or PN and can result in significant hypertriglyceridemia [18], even in the absence of propofol infusion syndrome [19].

### 4.1. Complications Associated with Hypertriglyceridemia and Caloric Overfeeding

Those with conditions susceptible to impaired exogenous lipid clearance due to decreased lipoprotein lipase activity (e.g., diabetes mellitus, pancreatitis, and renal failure); liver failure; or a history of hyperlipidemia, obesity, corticosteroid therapy, human immunodeficiency virus disease, or multisystem organ failure are at risk for hypertriglyceridemia during the administration of intravenous lipid emulsion [20,21]. Higher doses of intravenous propofol (lipid emulsion) can result in hypertriglyceridemia as lipoprotein lipase becomes saturated at a serum triglyceride concentration of ~300 to 400 mg/dL resulting in non-enzymatic means of elimination (e.g., from first order kinetics to zero order kinetics) with the lipid emulsion and its remnants being eliminated via the reticuloendothelial system [21]. Excessive serum triglyceride concentrations and increased appearance of chylomicron-like remnants from intravenous lipid emulsion can result in reticuloendothelial system clogging, compromised immune function, as well as pancreatitis [21,22,23]. However, some patients at risk for impaired clearance may also require a lower dosage of propofol for effective sedation, which could potentially dampen the appearance of hypertriglyceridemia.

Caloric overfeeding is also an issue particularly when high doses of propofol are combined with calories derived from enteral or parenteral nutrition (Table 1) [2,6,12,15]. PN, which is comprised of 60% glucose, 20% lipid, and 20% protein, given at a total caloric intake equal to the estimated basal energy expenditure and then increased to 1.5 times the estimated basal energy expenditure in ventilator dependent patients significantly increased carbon dioxide production by 35% [24]; this could potentially impair ventilator weaning. Caloric overfeeding, especially when the carbohydrate or glucose intake is in excess of 4 to 5 mg/kg/min, can also result in hyperglycemia [25]. Caloric excess, in general, has been associated with poorer outcomes. In a retrospective analysis of 1171 ICU patients from a mixed medical/surgical/trauma population with an ICU length of stay greater than 4 days, increased mortality was associated with a total caloric intake that exceeded about 1.2 to 1.3 times the measured resting energy expenditure [26].

Another potential option to reduce caloric and triglyceride intake is to employ a 2% (20 mg/mL) propofol emulsion instead of 1% (10 mg/mL), which would result in delivery of half the amount of lipid emulsion at an equivalent propofol dose. The 2% propofol emulsion is available in Europe and other international countries but has not been approved for use by the Food and Drug Administration (FDA) in the United States. However, in response to the COVID-19 pandemic, the FDA has issued an emergency use authorization to permit the use of this unapproved product. Despite this authorization, many institutions in the United States do not have the 2% product.

These data provide sufficient evidence that patients who receive concurrent propofol and EN or PN may need to have their nutrition regimens adjusted to provide less calories to prevent overfeeding. Serum triglycerides should also be routinely monitored while the patient is receiving propofol therapy.

### 4.2. The Rationale and Dilemma for Providing Sufficient Protein Intake

Recent studies have indicated an association between improved mortality, shorter ventilation days, and shorter duration of ICU and hospital stays with increases in protein intake for critically ill patients [26,27,28,29,30]. This is particularly relevant for those critically ill patients with a prolonged ICU stay, such as those with multiple traumatic injuries and TBI [28]. It is recommended by the Society of Critical Care Medicine (American Society for Parenteral and Enteral Nutrition) guidelines that critically ill patients receive 1.2 to 2 g/kg/d [22]. However, they also indicate that certain critically ill subpopulations such as those with trauma, obesity, and those who require continuous renal replacement therapy may need a greater protein intake (e.g., 2 to 2.5 g/kg/d) [22,31,32,33,34,35]. In order to overcome the anabolic resistance associated with aging, a greater protein intake may be required for those older than 59 years of age compared to younger adults to achieve the same nitrogen balance [36]. However, it should be acknowledged that the current literature is lacking in randomized prospective clinical trials to ascertain exactly how much protein is required or if an improvement in nitrogen balance for various homogenous populations will result in improved clinical outcomes; all require further study.

Table 1 suggests that most studies provided less than 1.2 g/kg/d of protein, which is inadequate for critically ill patients. This reduced protein intake may have been due to the concept of reducing the infusion rate of EN to avoid overfeeding or for patient conditions requiring fluid restriction [10,15,37]. Since EN formulas have a fixed composition, the macronutrient components cannot be altered unlike PN. Thus, reducing the rate of the continuous EN formula will also reduce the protein intake. Patients with traumatic injuries and TBI are among the most likely populations to receive prolonged, high dose propofol therapy (Table 1) [5,6,9] and require a greater protein intake than many other critically ill patients [22,33]. Therefore, a simple reduction in EN rate to decrease calories will result in decreased protein intake which could potentially be detrimental to clinical outcomes. Patients with TBI tended to receive greater protein intakes than other ICU populations when receiving propofol (Table 1); however, average protein intakes were still less than target goals. The reason for this inadequacy in achieving ideal protein intake was likely related to multiple factors, including interruptions in EN due to various surgical, interventional, and diagnostic procedures [38], as well as an increased incidence of gastric feeding intolerance and a greater amount of tachyphylaxis to metoclopramide therapy when compared to those without TBI [39].

### 4.3. Strategies to Avoid Overfeeding with Calories and to Maintain or Improve Protein Intake

#### 4.3.1. Parenteral Nutrition

For those institutions where PN solutions are compounded by the pharmacy, it is easy to adjust the individual components to meet energy and protein requirements. A typical approach is to either omit or decrease the amount of lipid emulsion, particularly for those receiving high dose soybean-oil based propofol therapy. If lipid calories beyond what is provided with the propofol infusion are desired, a mixed oil lipid emulsion could be added to the parenteral nutrition solution to avoid excessive intakes of long-chain omega-6 fatty acids. This approach also gives the prescriber flexibility in avoiding excessive lipid intake as the propofol rate may be increased and decreased throughout the day as the patient is titrated to the target Richmond-Agitation Sedation Scale score of −2 for light sedation [40]. The clinician will also need to assess if the patient is receiving the appropriate caloric intake based on previously established targets. Depending on the target caloric intake, the amount of dextrose in the dextrose-amino acid PN solution may need to be increased or decreased accordingly. Dextrose contributes 3.4 kcal/g and amino acids are 4 kcal/g. Some practitioners might find it confusing that a 10% lipid emulsion (propofol) contributes 11 kcal/g (1.1 kcal/mL), whereas intravenous lipids (20% or 30%) used in compounding PN solutions contribute 10 kcal/g (2 or 3 kcal/mL, respectively). This is because fat contributes 9 kcal/g, which would render the caloric content from only the fat component for a 10% intravenous lipid emulsion solution at 0.9 kcal/mL. However, to produce a lipid emulsion, egg phosphatides are used as the emulsifying agent and glycerol is also added to make the solution isotonic. Both components also contribute calories amounting to ~0.2 kcal/mL or 2 kcal/g of the 10% lipid emulsion. Thus, the 10% intravenous lipid emulsion product will contribute 11 kcal/g or 1.1 kcal/mL.

It is recommended that the dextrose intake not exceed 4 to 5 mg/kg/minute amounting to 20 to 25 kcal/kg/d to avoid overfeeding complications [25,41]. The brain and other glucose-dependent tissues require ~130 g of glucose daily [42]. For surgical, trauma, and thermally injured patients, glucose consumption of the wound may require an additional glucose intake of ~80 to 150 g daily depending on the extent of the wound [43,44]. Thus, it is our current practice to provide at least ~200 g of dextrose daily for surgical, trauma, and thermally injured patients if the patient is not experiencing significant hyperglycemia.

Accomplishing these goals for the institutions that use multi-chamber bag PN solutions, particularly for those patients with high protein needs, may be more challenging. The recent expansion of available products to include a 10% dextrose/8% amino acid and 14% dextrose/8% amino acid solutions may facilitate ease in meeting these goals. For the institutions that only have fat-containing multi (three)-chamber bags, breaking the seal between only the glucose-amino acid compartments will only allow the fat free components of the PN solution to be used. Another alternative solution is to piggyback an amino acid solution via a separate central venous catheter port (or via a Y-site, albeit less desirable if a separate port is not available) to be co-infused with the multi-chamber bag PN preparation if greater protein intake is desired beyond what is available in the multi-chamber bag PN.

#### 4.3.2. Enteral Nutrition

Avoiding overfeeding and providing sufficient protein for those who receive enteral nutrition concurrently with an intravenous propofol infusion can be very challenging, especially for highly catabolic and critically ill patients. The primary challenge results from EN formulations that are only available with fixed components and the macronutrients cannot be altered unlike PN solutions. Adult EN formulas range from 1 kcal/mL to 2 kcal/mL and the protein content can range from ~34 g/L to 94 g/L. The macronutrient components of various EN formulations vary based on the population for its intended use such as “standard formulas” for malnourished and unstressed or mildly stressed patients or disease/condition-specific formulas for those who require volume restriction or have renal, hepatic, or pulmonary impairment/failure; elevated protein needs; diabetes/hyperglycemia; or obesity. Additionally, there are EN formulas fortified with “immune modulating” ingredients or predigested/elemental diets. Liquid and powder modular protein supplements are also commercially available.

To avoid overfeeding, some clinicians have recommended adjusting the EN regimen or reducing the rate of the EN formulation [10,11,15,37]. Others suggested the use of a high protein (20%) containing formula at a reduced rate but admitted that the patient may not achieve target energy and protein needs [15]. Unfortunately, the use of volume restricted (2 kcal/mL) EN formulas for patients requiring fluid restriction are low in protein content when used for patients with high protein requirements. Table 1 indicates that most studies examining EN regimens resulted in inadequate protein intakes and often provided less than 1.2 g/kg/d [7,10,11,12,14,15,16].

We have recently published our approach to this dilemma in 51 critically ill patients with multiple traumatic injuries with severe TBI or with isolated severe TBI who received high doses of propofol requiring caloric restriction [6]. Our approach entailed the use of a “very high protein” enteral formula (92 g protein/L, 1 kcal/mL) at a reduced rate along with supplemental 15, 30, or 45 g liquid protein boluses multiple times throughout the day. One exception to this enteral formula selection was for patients with greater severity of injury (estimated Injury Severity Score > 20), whereby an immune-modulating formula (94 g of protein/L and 1.5 kcal/mL) with an enteral glutamine supplement and liquid protein doses were given [45]. The liquid protein supplements should be diluted to half strength for ease in administration via a small bore feeding tube but can be effectively delivered at full strength when administered via a larger bore tube (e.g., nasogastric suction tube or gastrostomy) [46]. If the EN infusion rate did not provide enough daily volume of feeding to meet the recommended dietary intake for vitamins (i.e., 1000 mL or 1500 mL depending on the formula), a liquid multivitamin preparation was provided daily.

**Figure 1:**
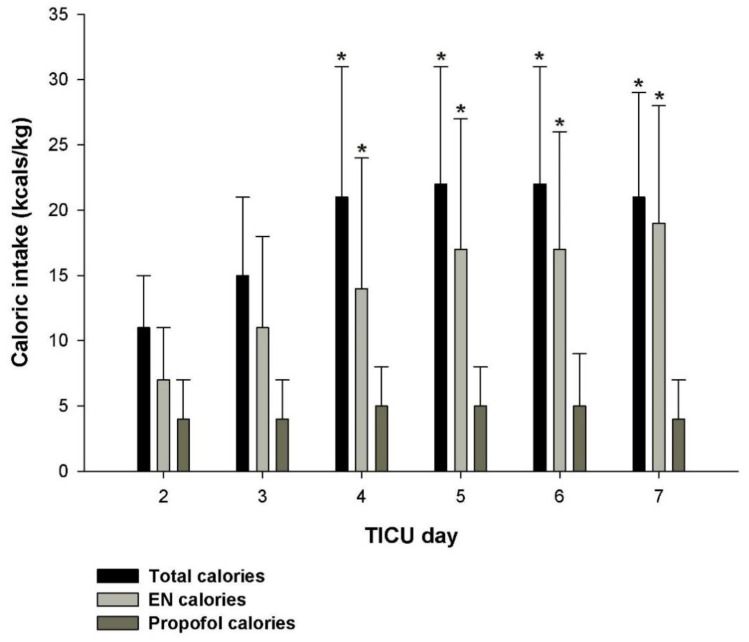
Total caloric intake, enteral nutrition calories, and propofol calories over days 2 to 7 for patients who received concurrent enteral nutrition and propofol [6]. One-way ANOVA indicated a significant difference in total caloric intake (*p* < 0.001) and enteral nutrition caloric intake (*p* < 0.001), but not propofol caloric intake (*p* = 0.076). * *p* < 0.05 from day 2. Day 1 represents a partial day upon admission to the TICU and was not included in the analysis. TICU, trauma intensive care unit.

The assigned target caloric intake (from both propofol and enteral nutrition) was 30 to 32 kcal/kg/d for those without obesity [33]. For those with obesity, a caloric goal of 22 to 25 kcal/kg ideal body weight (IBW)/d [35,47] was assigned. Patients received an average of 356 ± 243 kcals/d or 5 ± 3 kcal/kg/d from propofol but the caloric intake was widely variable among the patients and ranged from 1 kcal/kg/d to 15 kcal/kg/d. Caloric intake from EN ranged from 7 ± 4 kcal/kg/d on the first day of feeding to 16 ± 9 kcal/kg/d by the fifth day of EN. Caloric intake from large-volume dextrose-containing solutions averaged < 1 kcal/kg/d. The daily total caloric intakes using our approach to EN with concurrent propofol therapy during the study observation period are given in Figure 1.

**Figure 2:**
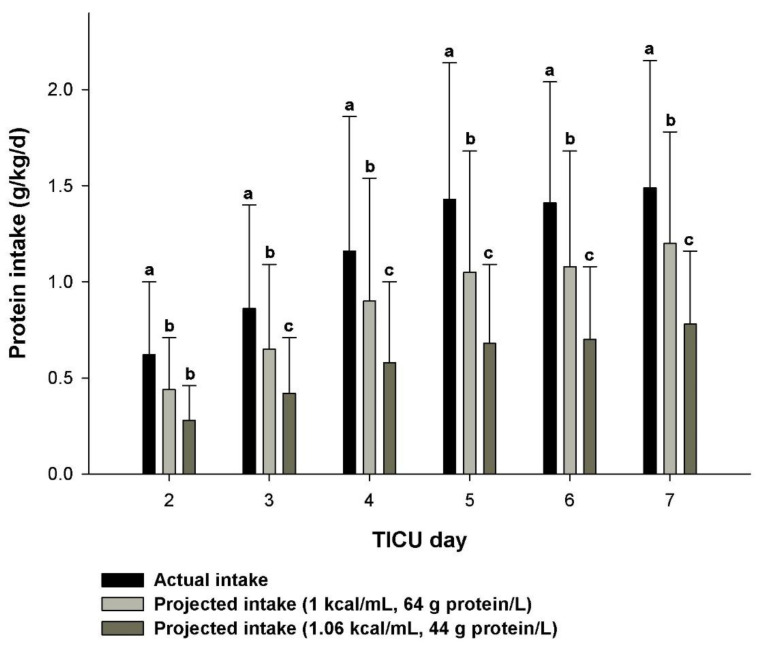
Actual protein intake from the modified EN regimen compared to projected protein intakes with “standard” formulas when given at an isocaloric intake as the modified EN regimen (*p* < 0.001) for patients who received concurrent enteral nutrition and propofol [6]. Daily protein intakes for each regimen that contain a different letter (e.g., a, b, or c) are significantly different from one another (*p* < 0.05). Day 1 represents a partial day upon admission to the TICU and was not included in the analysis. TICU, trauma intensive care unit.

Critically ill patients with traumatic injuries have high protein requirements of 2 to 2.5 g/kg/d [22,33] and providing a high protein intake with caloric restriction during propofol therapy is difficult. This is even more so for the obese patient who receives hypocaloric high protein nutrition therapy during propofol therapy and especially for those with a body mass index > 39.9 kg/m^2^ because it is recommended to provide 2.5 g/kg IBW/d of protein [22,31,32]. Figure 2 illustrates the advantage of increased delivery of protein with our approach as opposed to a simple reduction in EN feeding rate (without protein boluses). If the actual energy intake provided by use of this technique was met with either a “high protein” or standard enteral formula containing either 64 or 44 g of protein per liter, patients would have received 24% to 38% less protein despite receiving the same amount of calories. In addition, use of a “very high protein” containing EN formula with a reduced rate and supplemented with protein boluses resulted in the highest reported protein intake when compared with other studies (Table 1). Despite this improvement, we were still unable to meet protein goals for some patients because of interruptions in EN delivery due to feeding intolerance, multiple surgical procedures, inability to provide intravenous erythromycin (due to drug shortage, prolonged QTc interval, or significant drug-interaction) despite tachyphylaxis to metoclopramide therapy, and hemodynamic instability [38,39].

### 4.4. Case Studies

How this technique can be employed for a critically ill patient receiving high dose propofol therapy and requiring a high protein intake is illustrated in two case studies. It should be noted that our approach is based on our opinion supported by our study examining protein requirements to achieve nitrogen equilibrium for critically ill patients with TBI [33] and what is recommended in the 2016 SCCM-ASPEN guidelines [22]; however, other reasonable approaches to the nutritional management of these patients are possible. For both cases, consider a patient who is 1.78 m tall, weighs 90 kg, and has a BMI of 28.4 kg/m^2^. Both patients have multiple traumatic injuries including TBI and required mechanical ventilation and received an intravenous 1% propofol infusion at 25 mL/h. The propofol infusion provides about 7 kcals/kg/d.

The propofol calculations are as follows:

25 mL/h × 24 h = 600 mL

600 mL × 1.1 kcal/mL = 660 kcals

660 kcals/90 kg = 7.3 kcal/kg or about 7 kcal/kg

For both the PN and EN cases, we would opt for 30–32 kcal/kg/d and 2–2.5 g/kg/d as our target intakes [33]. Please note that the regimens provided in both cases represents the final regimen. Each regimen should be increased gradually over two or three days depending on the clinical status of the patient and tolerance of the regimen.

#### 4.4.1. Case Study 1 (PN)

In this scenario, the patient also had multiple abdominal injuries, developed an ileus, and could not be fed enterally. Since the gastrointestinal tract could not be used, PN was indicated. A PN regimen comprised of 330 g of dextrose, 210 g of amino acids, and 70 g of a 20% lipid emulsion would provide about 30 kcal/kg/d and 2.3 g/kg/d of protein. Glucose intake would be 2.5 mg/kg/min or 0.15 g/kg/h and does not exceed the recommended glucose infusion rates of 5 mg/kg/min or 0.3 g/kg/h.

The PN calculations are as follows:

330 g of dextrose × 3.4 kcals/g = 1122 kcals

330 g of dextrose × 1000 mg/g/90 kg/1440 min (in a day) = 2.5 mg/kg/min

330 g of dextrose/90 kg/24 h = 0.15 g/kg/h

210 g of amino acids/protein × 4 kcals/g = 840 kcals

70 g of 20% lipid emulsion × 10 kcal/g = 700 kcals

1122 kcals + 840 kcals + 700 kcals = 2662 total kcals

2662 kcals/90 kg = 29.6 or about 30 kcals/kg

210 g of amino acids (protein)/90 kg = 2.33 g/kg/d or about 2.3 g/kg/d

Since this patient was receiving propofol at 25 mL/h (7 kcals/kg/d), if the PN regimen was not adjusted for propofol calories then the patient would receive an excessive 37 kcal/kg/d when combining calorie content from both PN and propofol.

The combined PN and propofol calculations are as follows:

2662 kcals (from PN) + 660 kcals (from propofol) = 3322 kcals

3322 kcals/90 kg = 36.9 kcals/kg or about 37 kcals/kg

As mentioned previously, the general approach would be to omit the lipid emulsion from the PN regimen, which would comprise of 330 g of dextrose and 210 g of protein, providing 29 kcal/kg/d and 2.3 g/kg/d of protein when the propofol infusion calories are included.

The PN calculations are as follows:

330 g of dextrose × 3.4 kcals/g = 1122 kcals

210 g of amino acids/protein × 4 kcals/g = 840 kcals

1122 kcals + 840 kcals = 1962 total kcals

The combined PN and propofol calculations are as follows:

1962 kcals (from PN) + 660 kcals (from propofol) = 2622 kcals

2622 kcals/90 kg = 29.1 kcals or about 29 kcals/kg

In order to meet the target calorie goal of 30–32 kcal/kg/d, the dextrose component of the PN regimen could be increased to 350 g or the regimen could continue at the current rate depending on the clinical condition of the patient and the anticipated duration of the propofol infusion. If the dextrose content is increased, the final PN regimen of 350 g of dextrose and 210 g of protein would provide 23 kcal/kg/d and 2.3 g/kg/d of protein. However, when combined with the caloric content of intravenous propofol, this regimen would provide about 30 kcal/kg/d and 2.3 g/kg/d of protein.

The PN calculations are as follows:

350 g of dextrose × 3.4 kcals/g = 1190 kcals

350 g of dextrose × 1000 mg/g/90 kg/1440 min (in a day) = 2.7 mg/kg/min

350 g of dextrose/90 kg/24 h = 0.16 g/kg/h

210 g of amino acids/protein × 4 kcals/g = 840 kcals

1190 kcals + 840 kcals = 2030 total kcals

The combined PN and propofol calculations are as follows:

2030 kcals (from PN) + 660 kcals (from propofol) = 2690 kcals

2690 kcals/90 kg = 29.9 kcals or about 30 kcals/kg

#### 4.4.2. Case Study 2 (EN)

For the critically ill and ventilator-dependent patient with multiple traumatic injuries that can be fed enterally, a typical EN regimen would consist of a “high protein” formula (e.g., 1 kcal/mL and 64 g protein/L) at 100 mL/h plus 45 g of liquid protein once daily (100 total kcals per 15 g of protein from both protein and carbohydrate content in the supplement) as a bolus to provide 30 kcal/kg/d and 2.2 g/kg/d of proteins. Please note that the caloric intake of liquid protein supplements is variable depending on which product your institution provides.

The EN calculations are as follows:

100 mL/h × 24 h = 2400 mL

2400 mL × 1 kcal/mL = 2400 kcals

There are 100 kcals per 15 g of protein (contains carbohydrate and protein) for the liquid protein solution at our institution. Thus, solving for unknown X amount of kcals per 45 g of liquid protein

X kcals/45 g = 100 kcals/15 g

X kcals = (100 kcals/15 g) × 45 g = 6.67 kcals/g × 45 g = 300 kcals

2400 kcals (from EN formula) + 300 kcals from protein supplement = 2700 kcals

2700 kcals/90 kg = 30 kcal/kg

2400 mL/1000 mL/L = 2.4 L of EN

2.4 L × 64 g of protein/L = 153.6 or 154 g of protein

45 g protein (bolus) + 154 g of protein (EN formula) = 199 g protein

199 g protein/90 kg = 2.21 or about 2.2 g/kg

The combined EN and propofol calculations are as follows:

2700 kcals (from EN) + 660 kcals (from propofol) = 3360 kcals

3360 kcals/90 kg = 37.3 kcals or about 37 kcals/kg

The addition of propofol calories (7 kcal/kg/d) would provide a total of 37 kcal/kg/d, which puts this patient at risk for overfeeding complications.

Our approach would be to switch from a “high protein” formula to a “very high protein” formula (e.g., 1 kcal/mL and 92 g protein/L) at a reduced rate combined with supplemental liquid protein boluses. In this patient, changing to a “very high protein” regimen at 75 mL/h with 30 g of liquid protein supplement per day would provide 22 kcals/kg/d and 2.2 g/kg/d of protein.

The EN calculations are as follows:

75 mL/h × 24 h = 1800 mL

1800 mL × 1 kcal/mL = 1800 kcals

100 kcals per 15 g of protein (contains carbohydrate + protein), Solving for unknown amount of X calories from protein

X kcals/30 g = 100 kcals/15 g

X kcals = 30 g × 100 kcals/15 g = 30 g × 6.67 kcals/g = 200 kcals

1800 kcals (from EN formula) + 200 kcals from protein supplement = 2000 kcals

2000 kcals/90 kg = 22.2 kcal/kg or about 22 kcal/kg

1800 mL/1000 mL/L = 1.8 L of EN

1.8 L × 92 g of protein/L = 165.6 or 166 g of protein

30 g protein (bolus) + 166 g of protein (EN formula) = 196 g protein

196 g protein/90 kg = 2.18 or about 2.2 g/kg

With addition of propofol calories, this adjusted regimen would provide about 30 kcals/kg/d and 2.2 g/kg/d of protein.

The combined EN and propofol calculations are as follows:

2000 kcals (from EN and protein supplement) + 660 kcals (from propofol) =

2660 kcals

2660 kcals/90 kg = 29.6 kcals or about 30 kcals/kg

## 5. Conclusions

Providing a reduced calorie and high protein nutrition regimen to avoid overfeeding complications without severely compromising protein intake during continuous propofol therapy is challenging; however, it is possible for patients who require EN or PN if thought of creatively. Adjustments to the nutrition therapy is indicated as the patients’ propofol doses are escalated or reduced during combined propofol and nutrition therapy, especially if the duration for the propofol therapy is prolonged. Pharmacists and other health professionals engaged in nutrition support practice can utilize the techniques and considerations given in this review to facilitate safe and efficacious PN or EN for patients receiving propofol therapy.

## Figures and Tables

**Table 1:** Summary of reported calorie and protein intakes during concurrent propofol and nutrition therapy.

Author, Year	Patient Population	N	Nutrition Route	Calories from Nutrition, (kcals/d)	Calories from Propofol (kcals/d)	Total Calories (kcals/d)	% of Calories from Propofol	Duration of Propofol, Days	Protein Intake (g/kg/d)
Arruda, 2009	ICU ALI, ARDS	30	EN	-	356	-	-	-	-
Asa’ari, 2015 *	ICU	50	EN and PN	~1170	~130	~1300	10%	-	-
Bousie, 2016	ICU	101	EN and PN	~1400	230 (IQR 85–595)	~1600 kcals/d	14%	-	-
Buckley, 2021	Traumatic brain injury	51	EN	16 ± 9 Kcal/kg/d	356 ± 243 5 ± 3 Kcal/kg/d	22 ± 9 Kcal/kg/d	24%	6 ± 4	1.5 ± 0.7
Castro, 2020 *	COVID-19	39	EN	~1473	260	1733	15 ± 8%	8 ± 6	88% of goal
Charriere, 2017	ICU 1% or 2% propofol	687	EN	~1216	146 ± 117	1362 ± 811	17 ± 21% (first few days)	-	-
DeChicco, 1995 *	ICU	19	EN and PN	~1350	215 (range 79–535)	-	15% of BEE	-	-
Ferrie, 2013	Cardiac failure and ECMO	86	EN and PN	~1464	130 ± 184	1594 ± 628	8%	9 ± 4	58 ± 29 g/d
Hastings, 2018	ICU	325	EN and PN	~1231	119 (IQR 50–730)	~1350	8–10%	-	<1.2 for most pts
Ibarra, 2020 *	Trauma, Neurologic	26	EN	10	2 kcal/kg/d	12	18%	-	0.4
Rai, 2010	ICU Sepsis	43	EN	~1400	79 (range, 0–426)	~1500	5%	-	-
Richardson, 2018 *	ICU 1% propofol 2% propofol	79 75	EN EN	1031 1244	111 60	1142 1304	10% 5%	- -	61% (goal) 70% (goal)
Taylor, 2005	Trauma, Neurosurgical	85	EN	-	-	Goal: BEE X stress factors	19%	-	<90% goal for 51% of pts; <80% of goal for 21% of pts
Terblanche, 2020	CT-ICU 1% propofol 2% propofol	50 50	EN EN	13 ± 6 Kcal/kg/d 16 ± 5 Kcal/kg/d	353 (IQR 224 to 447) 185 (IQR 118 to 244)	16 ± 5 Kcal/kg/d 18 ± 7 Kcal/kg/d	14% 10%	- -	0.8 ± 0.4 0.9 ± 0.3

* Full paper unavailable or not published; data derived from the published abstract. ALI, acute lung injury; ARDS, adult respiratory distress syndrome; BEE, basal energy expenditure as determined by the Harris–Benedict equations; CT-ICU, cardiothoracic ICU; ECMO, extracorporeal membrane oxygenation; EN, enteral nutrition; ICU, intensive care unit; IQR, interquartile range; PN, parenteral nutrition; pts, patients.

## Data Availability

No new data were created or analyzed in this review article. Data sharing is not applicable to this article.
